# Prevalence and antibiotics resistance status of *Salmonella* in raw meat consumed in various areas of Lahore, Pakistan

**DOI:** 10.1038/s41598-023-49487-2

**Published:** 2023-12-14

**Authors:** Aiman Fatima, Maira Saleem, Shahid Nawaz, Linta Khalid, Saba Riaz, Imran Sajid

**Affiliations:** Institute of Microbiology and Molecular Genetics, University of the Punjab, Lahore, 54590 Pakistan

**Keywords:** Microbiology, Diseases

## Abstract

This study reports the prevalence and antibiotics resistance status of *Salmonella* detected in raw meat from Lahore, Pakistan. Overall, N = 111 meat samples, were collected from local markets. *Salmonella* was recovered from 57 (51.35%) samples, including 45.83% of poultry, 60% of buffalo, 64.28% of cow, and 60% of goat meat samples. The predominant *Salmonella* strains were *Salmonella enterica* serovars; Typhimurium (45.4%), Typhi (27.2%), and Enteritidis (18.1%), identified by VITEK system and 16S rRNA gene sequencing. The isolates exhibited high resistance to Erythromycin (100%), Cefepime (98.24%), Colistin (94.73%), Azithromycin (92.98%), Tetracycline (87.71%), Polymyxin B (84.21%), Ciprofloxacin (84.21%), Trimethoprim-Sulfamethoxazole (80.70%), Nalidixic Acid (80.70%), Kanamycin (78.94%), Chloramphenicol (77.19%), Streptomycin (71.92%) and Ampicillin (64.91%). While the isolates exhibited more susceptibility to Meropenem (75.43%) and Amikacin (73.68%). N = 8 strains were designated as Multidrug Resistant (MDR) and N = 3 as Extensively Drug-Resistant (XDR) *Salmonella*. The PCR-based detection of resistance genes revealed the presence of *bla*_TEM-1_ gene (100%), *catA1* gene (64%), and *gyrA* gene (18%). The whole genome sequencing (WGS) of two selected strains and subsequent downstream analysis confirmed the strains as MDR and XDR *Salmonella enterica* serovar Typhi. The study showed that raw meat consumed in Lahore carries a significantly high number of drug-resistant *Salmonella.*

## Introduction

Foodborne diseases (FBDs) are a persistent threat to global health. Approximately one in ten people get infected with contaminated food, and 33 million lives are lost each year around the world^1^. *Salmonella enterica* is a widespread zoonotic foodborne pathogen which is a serious hindrance to socioeconomic progress globally mainly in developing countries^2^. The infections caused by *S. enterica* continue to plague both developed and developing countries but frequently result in high mortality mostly in developing countries. Some of its serovars, such as *S. enteritidis*, *S. typhimurium*, *S. typhi*, and *S. paratyphi*, are the cause of serious health problems in humans^3^. The animal-derived foods are the most common carriers of FBDs caused by *S. enterica*^4^. The infection occurs by consuming contaminated poultry meat, mutton, beef, and pork. The raw meat becomes contaminated at different stages during animal skinning, slaughtering, evisceration, transport, and meat cutting in butcher shops at local markets^2^.

Being a tropical country, the ambient temperature in Pakistan remains favorable for the growth of food spoilage bacteria, which makes the meat processed in unhygienic conditions unsafe for human intake. Goat, sheep, cow, buffalo, camel, and poultry meat are usually consumed as a protein diet in the country^5^. Some factors are responsible for the transmission of *Salmonella* in humans, such as cross-contamination during meat processing, poor hygiene practices of butchers, improper storage of meat at markets, intake of undercooked or raw meat, insufficient refrigeration and reheating of prepared meat at homes, and a long gap between cooking and eating meat. Due to the ubiquitous nature of *Salmonella*, its control in food is difficult. Various serotypes of *Salmonella* have been found in food-producing animals, and many of them are usually excreted in the feces of apparently healthy animal^6,7^.

In animal husbandry, the increased use of antibiotics for growth promotion, therapeutics, prophylaxis, and metaphylaxis has led to the emergence of antibiotic resistance in commensal, opportunistic, and normal flora of food-producing animals. The vigorous and continuous use of antibiotics in an environment generates a selection pressure that helps the antibiotic-resistant bacteria to survive and access the food chain. Contaminated raw meat is the main source of antibiotic-resistant *S. enterica* infections in humans^3^. Many antibiotic-resistant serotypes of *Salmonella* are notorious for disturbing the food chain^1^. Another serious concern for public health is the transmission of multidrug-resistant (MDR) and extensively drug-resistant (XDR) *Salmonella* through the consumption of contaminated food. Several studies have reported the isolation of MDR *Salmonella* strains that were also resistant to clinically important antibiotics such as Fluoroquinolones, third-generation Cephalosporins, and Carbapenems, which is now considered as an emerging issue around the world^2,6^.

In order to monitor and control antimicrobial resistance (AMR), many developed countries use a regular AMR monitoring and surveillance system, for instance, National Antimicrobial Resistance Monitoring Systems (NARMS) in the United States^8^. On the other hand, due to the inadequacy of surveillance networks, accurate diagnostics, and laboratory capacity, there is limited monitoring and surveillance system in developing countries, such as Pakistan^9,10^. Over the last few years, several studies conducted in Pakistan were focused on checking the prevalence and antibiotic resistance of *Salmonella* in raw poultry meat^11^, however, limited data is available for raw mutton and beef^2^. Also, a few studies have reported the detection and incidence of antibiotic resistance genes in *Salmonella* isolated from raw meat in Pakistan. In this context, the present study was designed to evaluate the prevalence, antibiotic resistance pattern, and screening resistance genes in *Salmonella* serovars recovered from raw (mutton, beef, and poultry) meat samples collected from different markets in Lahore, Pakistan.

## Results

### Recovery of *Salmonella* from different types of meat samples

**Figure 1:**
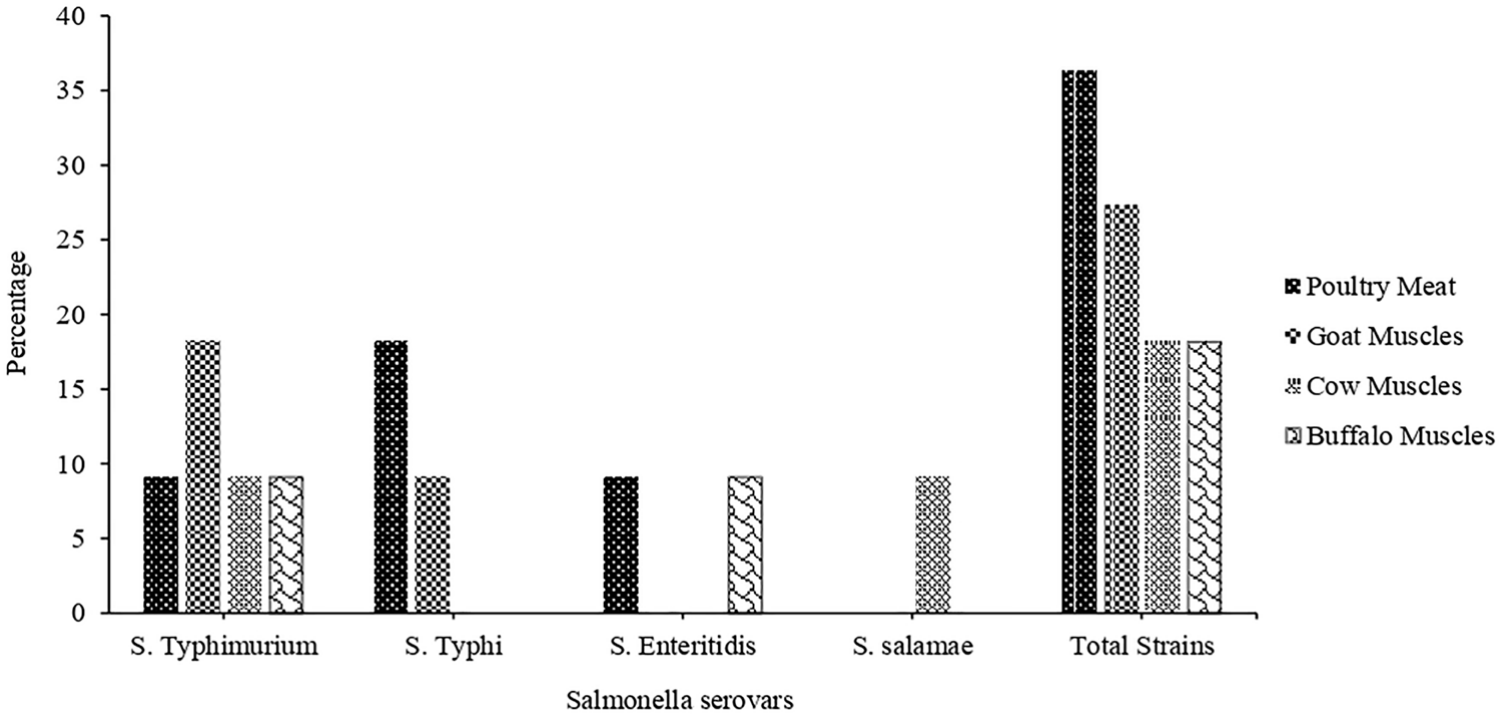
Prevalence of *S. typhimurium*, *S. typhi*, *S. enteritidis*, and *S. salamae* in different meat samples collected at local markets in Lahore, Pakistan.

Out of 111 different types of raw meat samples, *Salmonella* was recovered from 57 (51.3%) samples. The number of *Salmonella* isolates recovered from 72 poultry samples was 33 (45.83%), while the numbers of isolates from cow, buffalo, and goat meat were 9 (64.28%), 6 (60%) and 9 (60%), respectively. The poultry samples were subdivided into poultry muscles, wings, heart, liver, and gizzard. The percentage of recovery of *Salmonella* from poultry liver, poultry heart, poultry wing, poultry muscles, and poultry gizzard was 55.5% (10/18), 45% (9/20), 40% (4/10), 36.8% (7/19), and 60% (3/5), respectively (Table 1). Among these isolates, *Salmonella enterica* subsp. *enterica* serovars Typhimurium (45.4%), Typhi (27.2%), Enteritidis (18.1%), and *Salmonella enterica* subsp. *salamae* (9.09%) were more prevalent (Fig. 1). The identification of these strains was performed by VITEK 2 system that differentiated *Salmonella* from other bacteria having similar morphological growth characteristics such as *Proteus*. In the biochemical characterization, all these strains were catalase + ve, oxidase − ve, indole − ve, MR + ve, VP − ve, urease − ve, citrate + ve, phenylalanine deaminase test − ve, alkaline slant/acidic butt/H_2_S + ve in TSI test, and motility + ve in SIM test. The 16S rRNA gene sequence data for two strains (PG2 and CM4) was submitted to NCBI Genbank with accession numbers OP566900 (PG2) and OP604276 (CM4). The recovery rate of *Salmonella* was high in cow (64.28%), buffalo (60%), and goat (60%) meat as well as in poultry gizzard (60%) and liver (55.5%) (Table 1). *Salmonella* serovars’s prevalence in cow, buffalo, goat, and poultry meat is illustrated in Fig. 1.

**Table 1:** Recovery rate of *Salmonella* in various meat sources sold at local markets in Lahore, Pakistan.

Type of meat	Cow meat	Buffalo meat	Goat meat	Poultry meat				
				Gizzard	Liver	Heart	Wing	Muscle
Positive/total samples	9/14	6/10	9/15	3/5	10/18	9/20	4/10	7/19
Percentages	64.28%	60%	60%	60%	55.55%	45%	40%	36.84%

### MPN values of *Salmonella* from meat samples

In the majority of tested samples, the Most Probable Number (MPN) of *Salmonella* per milliliter of the sample was > 11, with 4.2 as the lowest number of *Salmonella* in the sample, which means that more than 11 most probable number or viable numbers of *Salmonella* were present in every sample. Some samples had *Salmonella* loads in the range of 1.2–11 MPN/ml, and a few had contaminated values in the range of 0.03–1.2 MPN/ml (Supplementary Table S2).

### Antibiotics resistance pattern of *Salmonella* isolates

**Figure 2:**
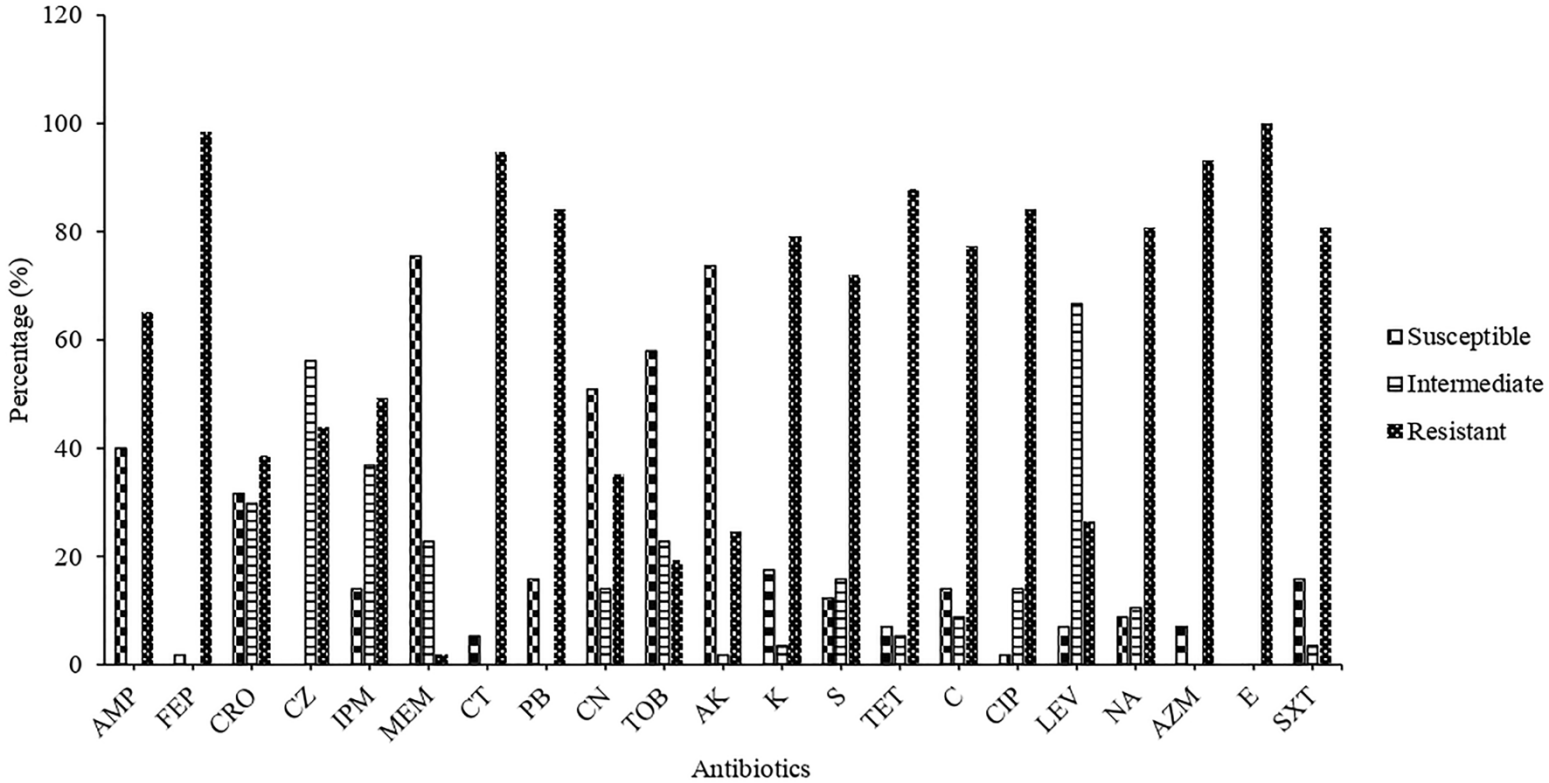
Antibiotics resistance pattern of *Salmonella* isolates recovered from different raw meat sources in Lahore, Pakistan.

The antibiotic resistance pattern of *Salmonella* strains to 21 different antibiotics has been summarized in Table 2 and Fig. 2. The strains were designated as “susceptible”, “intermediate”, and “resistant” by following the instruction of the Clinical and Laboratory Standards Institute (CLSI 2020). The highest resistance was recorded against Erythromycin (100%), which was followed by Cefepime (98.24%), Colistin (94.73%), Azithromycin (92.98%), Tetracycline (87.71%), Polymyxin B (84.21%), Ciprofloxacin (84.21%), Nalidixic acid (80.70%), Sulphamethoxazole-Trimethoprim (80.70%), Kanamycin (78.94%), Chloramphenicol (77.19%), Streptomycin (71.92%), Ampicillin (64.91%) and Imipenem (49.12%) (Table 2). In comparison to all the antibiotics, the resistance against Meropenem was less (1.75%). Moreover, Amikacin in the aminoglycosides group was the most effective antibiotic, with 73.68% of *Salmonella* strains susceptible to it. However, none of the *Salmonella* strains could be found to be sensitive to all antibiotics used in this study. While following the CDC criteria, *Salmonella* isolates phenotypically resistant to Ampicillin, Chloramphenicol, and Sulphamethoxazole-Trimethoprim were designated as MDR strains, and *Salmonella* strains resistant to the above-mentioned antibiotics along with third-generation Cephalosporin and Fluoroquinolones were designated as XDR strains. Based on the antibiotics resistance pattern, 8 MDR and 3 XDR *Salmonella* strains were recovered from raw meat in this study, which were only susceptible to Meropenem, making it the only effective treatment option for the infections caused by MDR and XDR *Salmonella* Spp. (Supplementary Table S3).

**Table 2:** *Salmonella* isolates susceptible, intermediate and resistant to various antibiotics.

Class/group of antibiotics	Antibiotics	Abbreviation	Disc potency (μg)	Susceptible (%)	Intermediate (%)	Resistant (%)
Penicillin	Ampicillin	AMP	10	20 (40)	0	37 (64.91)
Cephalosporin	Cefepime	FEP	30	01 (1.75)	0	56 (98.24)
	Ceftriaxone	CRO	30	18 (31.57)	17 (29.82)	22 (38.59)
	Ceftazidime	CZ	30	0	32 (56.14)	25 (43.85)
Carbapenems	Imipenem	IMP	10	08 (14.03)	21 (36.84)	28 (49.12)
	Meropenem	MEM	10	43 (75.43)	13 (22.80)	01 (1.75)
Lipopeptides	Colistin	CT	10	03 (5.26)	0	54 (94.73)
	Polymyxin B	PB	300U	09 (15.78)	0	48 (84.21)
Aminoglycosides	Gentamicin	CN	10	29 (50.87)	08 (14.03)	20 (35.08)
	Tobramycin	TOB	10	33 (57.89)	13 (22.80)	11 (19.29)
	Amikacin	AK	30	42 (73.68)	01 (1.75)	14 (24.56)
	Kanamycin	K	30	10 (17.54)	02 (3.50)	45 (78.94)
	Streptomycin	S	10	07 (12.28)	09 (15.78)	41 (71.92)
Macrolides	Azithromycin	AZM	15	04 (7.01)	0	53 (92.98)
	Erythromycin	E	15	0	0	57 (100)
Tetracyclines	Tetracycline	TET	30	04 (7.01)	03 (5.26)	50 (87.71)
Quinolones and fluoroquinolones	Ciprofloxacin	CIP	05	01 (1.75)	08 (14.03)	48 (84.21)
	Levofloxacin	LEV	05	04 (7.01)	38 (66.66)	15 (26.31)
	Nalidixic Acid	NA	30	05 (8.77)	06 (10.52)	46 (80.70)
Folate pathway antagonists	Sulfamethoxazole-Trimethoprim	SXT	1.25/23.75	09 (15.78)	02 (3.50)	46 (80.70)
Phenicols	Chloramphenicol	C	30	08 (14.03)	05 (8.77)	44 (77.19)

**Figure 3:**
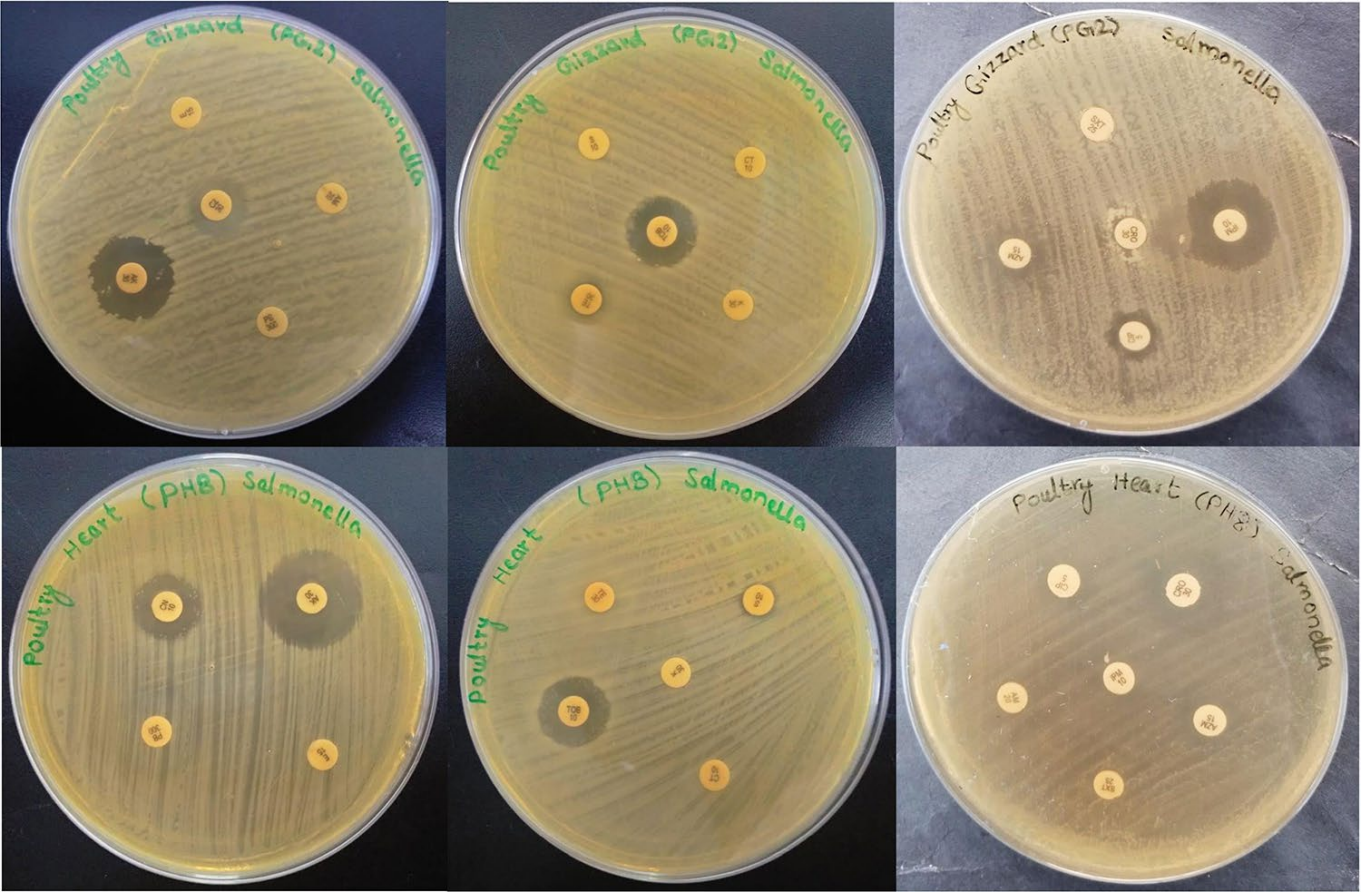
Antibiotic resistance pattern of an MDR and an XDR *Salmonella* strain recovered from raw meat.

Figure 3 demonstrates an XDR and an MDR *Salmonella* strain showing resistance to various antibiotics used in this study.

### PCR-based detection of antibiotics resistance genes

**Figure 4:**
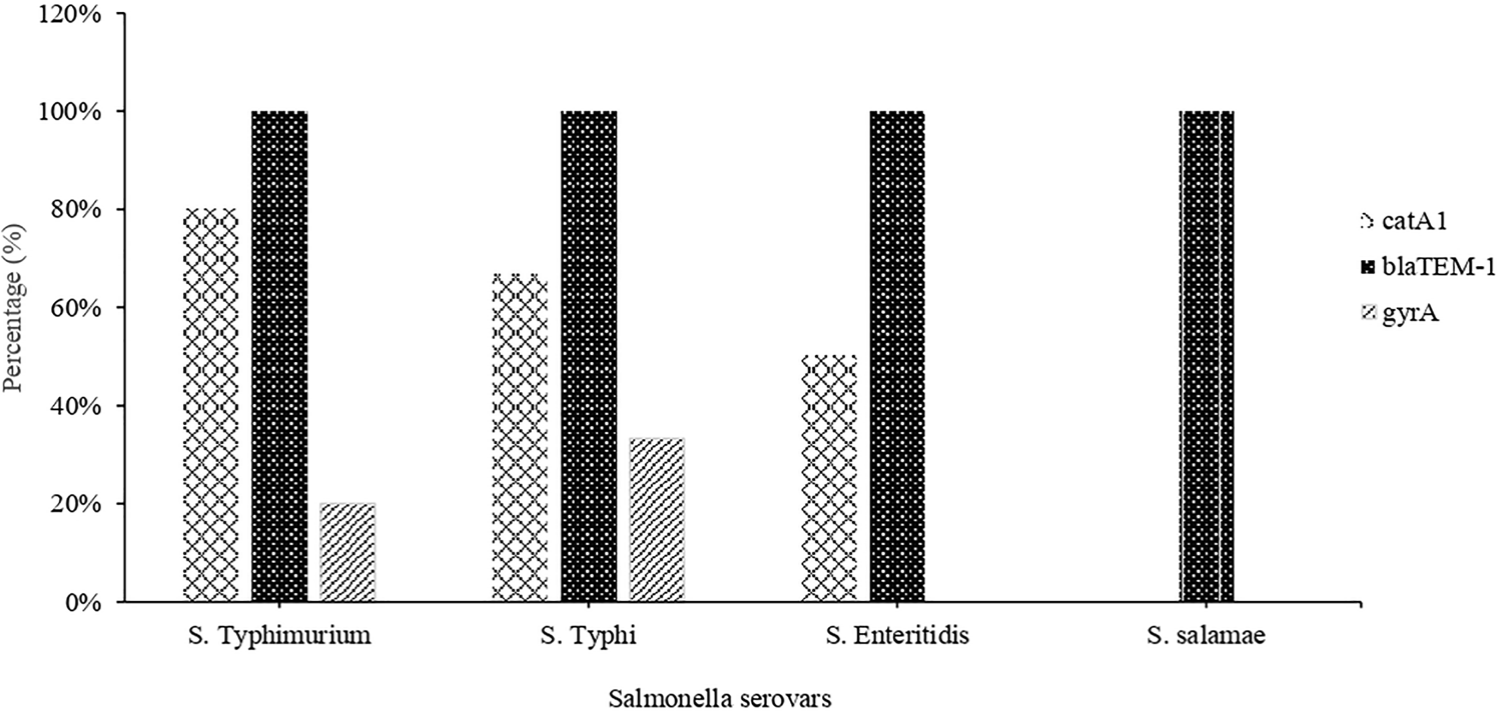
Prevalence of antibiotic-resistance genes in *Salmonella* isolates recovered from raw meat in Lahore, Pakistan.

**Figure 5:**
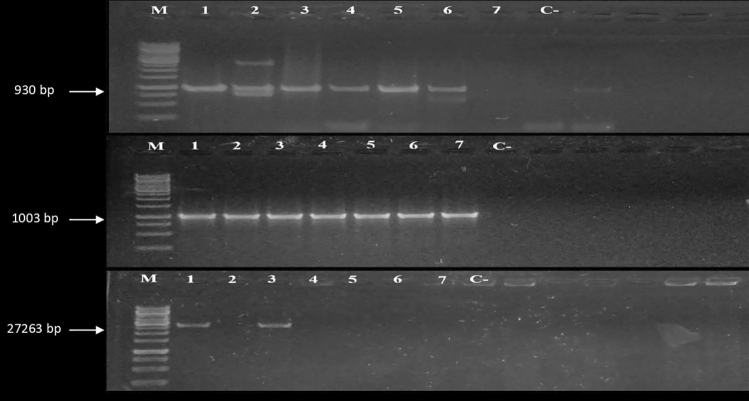
PCR-based detection of *blaTEM-1* (930 bp), *catA1* (1003 bp), and *gyrA* (2726 bp) genes. Lane M = 1 kb Ladder, C −  = Negative control, and Gel concentration = 1%.

The resistant *Salmonella* isolates were screened for three antibiotic resistance genes (*bla*_TEM-1_, *catA1*, *gyrA*) through PCR. The results in Fig. 4 and Supplementary Table S3 indicate that the *gyrA* gene was only detected in two resistant serovars, i.e. *S. typhi* and *S. typhimurium*. While *catA1* gene was found in seven resistant strains belonging to three groups of serovars, such as *S. typhi*, *S. typhimurium*, and *S. enteritidis*. On the other hand, each MDR and XDR *Salmonella* strain harbored the *bla*_TEM-1_ gene. Figure 4 summarizes the prevalence of antibiotic-resistance genes in different *Salmonella* serovars. The results of amplified products of *bla*_TEM-1_, *catA1*, and *gyrA* genes after gel electrophoresis are represented in Fig. 5.

### Whole genome sequence (WGS) analysis

**Figure 6:**
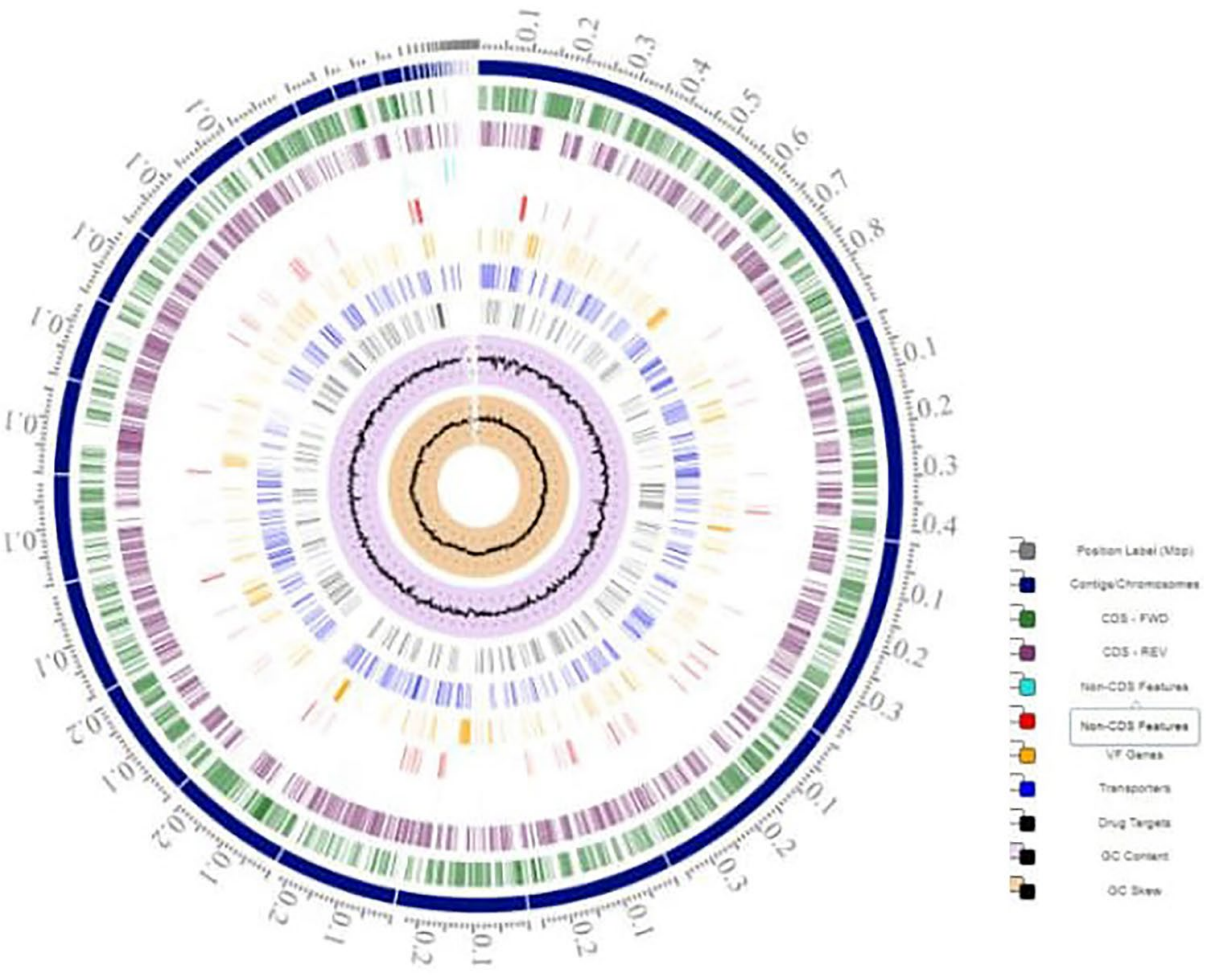
Circular view of genome features of *Salmonella enterica* MDR1-PH8 through BV-BRC (Version 3.28.21).

**Figure 7:**
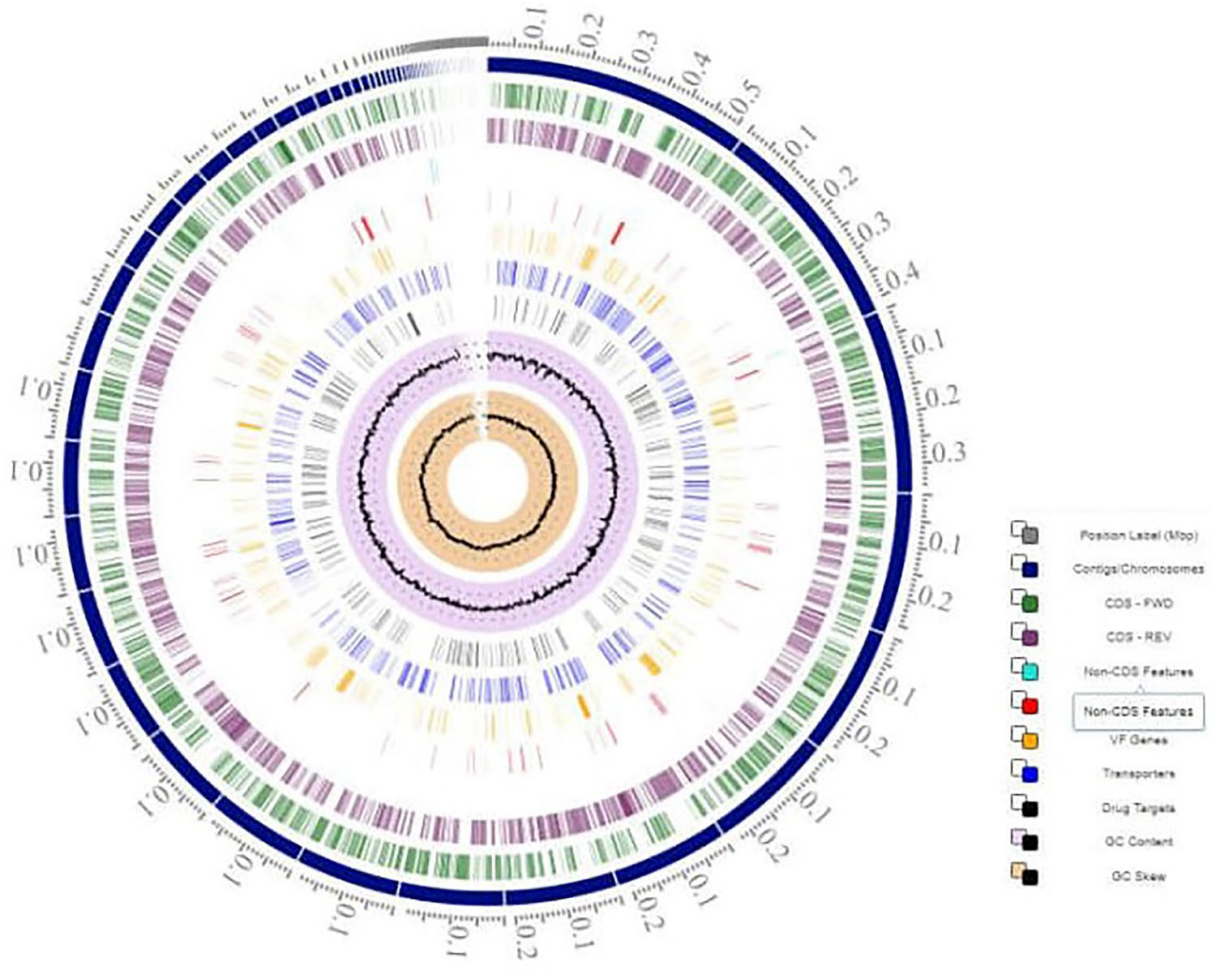
Circular view of genome features of *Salmonella enterica* XDR1-PG2 through BV-BRC (Version 3.28.21).

The assembled genome of *Salmonella enterica* strain MDR1-PH8 is composed of 71 Contigs (N50 = 217,896) sizing around 4,836,989 bp with GC content of 52% (Fig. 6). The assembled genome of *Salmonella enterica* strain XDR1-PG2 is composed of 42 Contigs (N50 = 244,952) sizing around 4,772,161 bp with GC content of 52% (Fig. 7). The genome annotations performed by PATRIC (BV-BRC) are summarized in the Table 3. The strain MDR1-PH8 exhibited ArthoANIu value % = 99.96, while the strain XDR1-PG2 exhibited ArthoANIu value% = 99.98 with the reference *Salmonella enterica* serovar Typhi (NCBI Taxonomy ID: 90370). The whole genome sequencing (WGS) and subsequent sequence analysis of the strains including genome annotations and average nucleotide identity (ANI) calculation with the reference *Salmonella eneterica* strain confirmed the identity of both of these strains as *Salmonella enterica* serovar Typhi. The genome sequence data of both the strains is submitted to NCBI, SRA, under the BioProject: PRJNA985576, GenBank, accession numbers JAUDSV000000000 and JAUDPT000000000, respectively.

**Table 3:** The genomic annotation of an MDR and XDR *Salmonella typhi* strains after whole genome sequencing analysis.

Genome/annotation statistics	*Salmonella enterica* MDR1-PH8	*Salmonella enterica* XDR1-PG2
Genome length (bp)	4,836,989	4,772,161
Contigs	71	42
Contig L50	8	6
Contig N50	217,896	244,952
GC content	52.01918	52.012577
tRNA	71	71
rRNA	10	9
CDS	5087	4995
CDS ratio	1.0516874	1.0466956
Hypothetical CDS	622	587
Hypothetical CDS ratio	0.28523687	0.2812813
PLFAM CDS	4954	4875
PLFAM CDS ratio	0.9738549	0.975976

## Discussion

*Salmonella* is one of the leading food-borne pathogens that cause food-borne illnesses worldwide. Animal-derived food such as meat can serve as a mode of transmission of *Salmonella* to humans^4^. Lahore is the 2nd largest and a highly populated city in Pakistan. Due to this high population, the demand for food, especially meat and meat products, is also very high. Such circumstances could result in poor food processing and management^12^. This study aimed to determine the prevalence of *Salmonella* spp. in different raw meat samples collected at different local markets in Lahore. The prevalence of *Salmonella* in poultry meat was found to be 45.83%, which is similar to the percentage obtained in other studies^10,13^. In poultry meat, higher prevalence of *Salmonella* were found in poultry gizzard (60%), followed by poultry liver (55.5%), heart (45%), wing (40%), and muscles (36.84%). These results contradicted the research findings of Sodagari et al.^14^, in which prevalence was recorded as 8.3% in poultry gizzard, 21.6% in poultry liver, and 14.1% in poultry heart. In this study, the prevalence of *Salmonella* in buffalo, cow, and goat meat was 60%, 64.28%, and 60%, respectively, which is higher as compared to other studies^2,3,15^. In general, it was observed, *S. typhimurium*, *S. typhi*, and *S. enteritidis* were the dominant serovars isolated from raw meat. However, comparing the prevalence of *Salmonella* serovars from different studies could be challenging due to the difference in geographical locations, sampling seasons, nature of samples, isolation methods, and abattoirs and retail shops environment^16^. In comparison with other serovars, *S. typhimurium* had a high prevalence (45.5%) in our findings which is supported by the study of Khan et al.^17^. *S. typhi* is a human-specific serovar, and its detection in food shows hygiene failure and improper handling. Very few studies have reported the prevalence of *S. typhi* in raw meat^18,19^. However, *S. typhi* prevalence was 27.2% in highly resistant strains isolated from raw meat in this study. Another dominant serovar was *S. enteritidis* (18.1%); however, other studies have reported a high prevalence than the findings in this study^2,20^. In this study, VITEK system was applied to avoid the misidentification of recovered strains as *Salmonella*, as some other bacteria such as *Proteus* and *Citrobacter* have similar growth characteristics and may cause false identification^21,22^. Many studies have described that VITEK system is a reliable method for the correct identification of Gram-negative rods^23^. The WGS results and phylogenetic tree analysis confirmed the identity of the strains as *Salomonella enterica* serovar Typhi. These results verified the original findings regarding the identity of the strains as *Salmonella* by VITEK characterization and biochemical analysis.

The study also provides quantitative data on the contamination of raw meat with *Salmonella* in Lahore city. The most probable number (MPN) of *Salmonella* per liter/g of meat was obtained mostly in the range of > 11 or 1.2–11, demonstrating a high load of *Salmonella* in raw meat, unlike the study of Yang et al.^24^, in which MPN/g was found in the range of 0.3–10. Another study conducted in Pakistan reported 3.6 MPN/g of *Salmonella*, which also shows low numbers than the current study^17^. Different raw meat samples heavily contaminated with *Salmonella* indicate poor hygiene situations and practices by butchers in different meat processing stages, which is also indicated in other studies^25,26^. Moreover, retail meat shops also lack refrigerators for meat storage which can promote the growth of *Salmonella* in meat. Another possibility of the presence of *Salmonella* in cattle could be the ingestion of contaminated food by animals or grazing those plants which might have been irrigated with unprocessed effluents containing fecal matter as fertilizer^27^. The risk of *Salmonella*-induced foodborne diseases is higher in developing countries as compared to developed countries^6^.

The *Salmonella* serovars recovered from raw meat in the present study were highly resistant to antibiotics, such as Ampicillin (64.91%), Erythromycin (100%), Tetracycline (87.71%), Colistin (94.73%) and Ciprofloxacin (84.21%), which are widely used as growth promoters and therapeutics in the livestock and poultry industry in Pakistan. This overuse and misuse of antibiotics have caused the transmission of drug-resistant *Salmonella* to humans via the food chain, which has a potentially negative impact on human health^28,29^. In our study, 21 different antibiotics were tested against *Salmonella* isolates, and high resistance was recorded against several clinically important antibiotics, such as Cefepime (98.24%), Azithromycin (92.98%), Ciprofloxacin (84.21%), Nalidixic Acid (80.70%), Sulphamethoxazole-Trimethoprim (80.70%), Chloramphenicol (77.19%), Ampicillin (64.91%) and Imipenem (49.12%) (Table 2, Fig. 2). The resistance to Ampicillin, Chloramphenicol, Sulphamethoxazole, Ceftriaxone and Ciprofloxacin in *Salmonella* is threatening as Carbapenems remain the last drug of choice to treat invasive *Salmonella* infections^3,30^. However, high resistance to Imipenem (49.12%) was found in this study. The previous studies conducted on raw meat had not reported such a high-level resistance to Azithromycin (92.98%) as detected in this study. The possible reason is the widespread use of Azithromycin for COVID-19 treatment in Pakistan^31^. Interestingly, resistance to antibiotics, which are not commonly used in animal and poultry farming demonstrates the significance of other resistance acquiring mechanisms and sources, for instance, horizontal gene transfer, cross-resistance in the same antibiotic group, animal feed contaminated with resistant environmental bacteria, and antibiotics used to treat human infections^3^. Eleven *Salmonella* strains isolated in this study were reported as MDR and XDR *Salmonella*. This is in agreement with several recent studies, which have reported the presence of MDR and XDR *Salmonella* in raw meat worldwide, as well as in Pakistan^11,32,33^.

Different serovars of *Salmonella* can possess a number of different antibiotic-resistant genes. However, data about the detection of resistance genes in *Salmonella* isolated from raw meat is rare in Pakistan. In this study, three antibiotic resistance genes were detected in *Salmonella* strains recovered from raw meat. Given the PCR results, the *bla*_TEM-1_ gene was detected in all isolated resistant strains (100%), which is supported by Li et al.^16^ in their study by detecting a 100% prevalence of *bla*_TEM-1_ gene. The prevalence of the *catA1* gene in all resistant *Salmonella* strains was 63.6% in our study, predominantly detected in *S. typhimurium* (Fig. 4). As compared to our study, the prevalence is low (47.5%) in the findings of Li et al.^34^. On the other hand, the *gyrA* gene was detected in only XDR *Salmonella* strains with a prevalence of 18.1% (Table S3, Fig. 4), in accordance with a previous study that detected the *gyrA* gene in 20% of *Salmonella* isolates^35^. The whole genome sequence (WGS) analysis, specifically the ANI values of the representative MDR-PH8 and XDR-PG2 strains up to 99.96% and 99.98% respectively with the reference *Salmonella enterica* serovar Typhi further confirmed the identity of these strains as *Salmonella enterica* serovar Typhi. The genome size of both the sequenced strains and GC content up to 52% is typically in the range for Gram negative bacteria and the members of enterobacteriaceae. Similarly the genome annotations, the number of CDS and subsystems details generally resemble to that of the *Salmonella* strains, which helped to confirm the identity of the isolated strains in this study as *Salmonella enterica* serovar Typhi. Overall this study provides comprehensive and useful data regarding the antibiotic resistance status of *Salmonella* isolated from raw meat in Lahore.

## Methods

### Sample collection

A total of 111 raw meat samples of various categories including poultry meat, mutton, and beef were collected between September 2021 and August 2022. The samples were randomly collected from meat shops located in eleven different local markets in Lahore (including Faisal Town market, Johar Town market, Wafaqi Market, Main Defense market, Township market, Model Town market, Barkat Market, Gulberg market, Allama Iqbal Town market, Samanabad Market, and Shahdara market), and the sampling was carried out once a week. The raw meat samples from different sources included poultry muscles (19), poultry heart (20), poultry liver (18), poultry gizzard (5), poultry wings (10), buffalo meat (10), cow meat (14), and goat meat (15) [Total n = 111]. Sterile plastic bags were used to collect the samples aseptically, which were then labelled and transported in an icebox to the research labs at the Institute of Microbiology and Molecular Genetics within 4–6 h for further processing.

### Sample processing for the isolation of *Salmonella*

Each of the sample was processed in three steps, i.e. pre-enrichment, selective enrichment, and isolation on selective media. For pre-enrichment, 10 g of each raw meat sample was homogenized and aseptically transferred into a flask containing 90 ml sterile Buffered Peptone Water (BPW, Oxoid CM1049) as a pre-enrichment media and incubated at 37 °C for 24 h. After overnight incubation, aliquots from pre-enrichment were transferred into a selective enrichment liquid medium at a ratio of 1:10, i.e. 1 ml of the cultured BPW was inoculated into each test tube containing 10 ml of Selenite-Cysteine Broth (Oxoid CM0699). The tubes were incubated at 37 °C for 24 h. The next day, a loopful from Selenite-Cystine Broth was sub-cultured onto Salmonella-Shigella (SS) agar (Liofilchem 610042), XLT-4 agar (Oxoid CM1061), and MacConkey agar (Oxoid CM0007) by using the streak plate method. These plates were incubated overnight at 37 °C. After incubation, the colonies with desired characteristics were selected and re-cultured to purify the bacteria from mixed cultures. The purified isolates were also cultured on blood agar (Oxoid CM0055).

### Identification of *Salmonella*

The colorless colonies with black centers on SS agar, black or red colonies with a black center on XLT-4 agar, colorless colonies on MacConkey agar, and white/grey non-swarming growth on blood agar were supposed to be *Salmonella* (as shown in Fig. S.1). The presumptive colonies of *Salmonella* were then analyzed by gram staining and biochemical characterization, which included the catalase test, oxidase test, indole test, Triple Sugar Iron (TSI) test, Sulfide Indole Motility (SIM) test, MR/VP tests, urease test, citrate utilization test, and phenylalanine deaminase test by following the protocols of International Organization for Standardization^36^.

### Strains identification by VITEK 2 compact system

The polystyrene tubes containing 3 ml saline solution were prepared, in which pure bacterial cultures were mixed to make 0.50–0.63 McFarland standard. For gram-negative antibiotic susceptibility (GN-AST), 145 µl of GN-ID McFarland solution was taken and mixed in another tube containing 3 ml saline solution. GN-ID and GN-AST cards were placed in the tubes respectively. Then the racks containing tubes and test cards were placed in the filling chamber of VITEK 2 compact system for loading samples. When the test cards had been filled, the racks were transferred to the other chamber for barcode scanning and cutting the filling straws from the cards, and incubating the cards. After logging into VITEK 2 software on the system, the cassette view and samples showing incomplete status were checked. The accession numbers of the samples were entered and the AST of these samples were linked with their IDs. The results were noted after 18 h.

### Enumeration of *Salmonella* using the MPN method

The three-tube three-dilution Most Probable Number (MPN) method was used to enumerate the load of *Salmonella* in each sample with some modifications in the protocol of the U.S. Department of Agriculture, Food Safety and Inspection Service (USDA–FSIS)^37^. For this purpose, a 25 g meat sample was homogenized, 225 ml of Buffered Peptone Water (BPW) was added, and incubated for 60 min at room temperature. After an hour, 10 ml of sample and BPW mixture was transferred into 3 empty sterile test tubes. Then, 1 ml and 0.1 ml aliquots of prepared homogenate were transferred into further 3, 3 test tubes having 9 ml of sterile BPW to make the first and second tenfold dilutions, respectively. All the inoculated tubes were incubated overnight at 37 °C. After incubation, a loopful from each tube was streaked onto SS agar and incubated at 37 °C for 24 h. The presumptive *Salmonella* colonies on each plate were subjected to biochemical testing. After confirmation of the number of positive tubes in each set, MPN/ml was calculated by using the USDA–FSIS MPN table^37^. The enumeration of *Salmonella* in each sample using the MPN technique was similar to the qualitative method used to isolate *Salmonella* in this study.

### Antibiotic susceptibility testing (AST)

In this study, the Kirby–Buyer disk diffusion susceptibility method was used to check the resistance and susceptibility of the *Salmonella* isolates to the antibiotics that are commonly used in animal husbandry and for treating *Salmonella* infections in humans, and also to detect the MDR and XDR *Salmonella* isolates based on their antibiotic resistance pattern. The few colonies of the identified bacterial isolates were picked and were inoculated in nutrient broth using sterile cotton swabs, after growth the inoculum was prepared by comparison with the 0.5 McFarland standard. With a fresh cotton swab soaked in this suspension, swabbing was done over the surface of Mueller–Hinton Agar (Oxoid CM0337), and the plates were left on bench top for a few minutes. Afterward, the antibiotic discs 6 mm in diameter (Bioanalyse) were placed and slightly pressed on the Mueller–Hinton Agar surface with a uniform space between them. These plates were kept at room temperature for 30 min and then incubated at 37 °C for 16–18 h. After incubation, the diameter of the zone of inhibition was measured in millimeters and evaluated using the Clinical and Laboratory Standards Institute (CLSI) recommendations for Enterobacterales^38^, and MDR and XDR *Salmonella* isolates were detected by following the criteria given by the Center for Disease Control and Prevention^39^. The antibiotics used in this study included; Ampicillin (20 μg), Cefepime (30 μg), Ceftriaxone (30 μg), Ceftazidime (30 μg), Imipenem (10 μg), Meropenem (10 μg), Colistin (10 μg), Polymyxin B (300 U), Gentamicin (30 μg), Tobramycin (10 μg), Amikacin (30 μg), Kanamycin (30 μg), Streptomycin (10 μg), Azithromycin (15 μg), Tetracycline (30 μg), Ciprofloxacin (5 μg), Levofloxacin (5 μg), Erythromycin (15 μg), Trimethoprim-Sulphamethoxazole (1.25/23.75 μg), Chloramphenicol (30 μg), and Nalidixic Acid (30 μg).

### DNA extraction and molecular identification of *Salmonella*

The GeneJET Genomic DNA Purification Kit (Thermo Scientific #K0721) was used to extract the DNA for PCR amplification as per the manufacturer’s instructions. Of all *Salmonella* isolates, only highly resistant *Salmonella* strains were selected for further molecular characterization based on their AST results. The oligonucleotide primers, i.e. F-AGAGTTTGATCMTGGCTCAG and R-GGTTACCTTGTTACGACTT (Synbio Technologies, USA) targeting the 16S rRNA gene (1465 bp), were used for the PCR-based identification of *Salmonella*. The PCR reaction mixture contained 25 μl of 2X Dream Taq Green Master Mix (Thermo Scientific™ #K1072), 18 μl of nuclease-free water (Thermo Scientific #R0581), 1.5 μl of both primers, and 4 μl of DNA template to make the final volume of 50 μl. The PCR reaction steps were as follows; initial denaturation at 95 °C for 5 min, then 30 cycles of denaturation at 95 °C for 30 s, annealing at 57 °C for 30 s, and extension at 72 °C for 30 s, which was followed by a final extension at 72 °C for 5 min. The negative control was made by replacing the DNA template with nuclease-free water in the PCR reaction mixture. After PCR, the obtained products were run on 1% agarose gel with GeneRuler 1 kb DNA ladder (Thermo Scientific #SM0311) and visualized under the UV-illuminator. The desired DNA bands were purified from the gel using the GeneJET Gel Extraction Kit (Thermo Scientific #K0691), which were then sent to BGI Tech, China for 16S rRNA gene sequencing. The BLASTn program available on the NCBI website (http://blast.ncbi.nlm.nih.gov/Blast.cgi) was used to perform a similarity search between the 16S rRNA gene sequencing results and already identified sequences found in the NCBI database.

### PCR based screening and detection of antibiotics resistance genes

The three antibiotic-resistance genes including *bla*_TEM-1_, *catA1*, and *gyrA* conferring resistance to ampicillin, chloramphenicol, and quinolones were detected through PCR. The PCR primer sequences and reaction conditions to amplify the target genes are tabulated in Supplementary Table S1. PCR amplification was processed in a 15 μl reaction mixture having 7.5 μl of 2× Dream Taq Green Master Mix (Thermo Scientific #K1072), 3.5 μl of nuclease-free water (Thermo Scientific #R0581), 0.5 μl of both primers, and 3 μl of DNA template. PCR reaction steps programmed on the thermocycler were: initial denaturation at 94 °C for 3 min, then 35 cycles of denaturation at 94 °C for 30 s, annealing (see Table S1), and extension at 72 °C for 1 min/kb (see Table S1 for amplicon size), and final extension at 72 °C for 7 min. A drug susceptible *Salmonella* isolate was used as a negative control in these PCR reactions. The PCR products were run on 1% agarose gel with GeneRuler 1 kb DNA ladder (Thermo Scientific #SM0311) and visualized under the UV-illuminator.

### Whole genome sequencing (WGS)

The whole genome sequencing (WGS), of two selected *Salmonella* strains including, MDR1-PH8 and XDR1-PG2 was performed. The pure strains were sent to MicrobesNG (United Kingdom) for sequencing. There, the genomic DNA was isolated and quality assessment followed by library preparation took place. After DNA was fragmented and ligated to Illumina adapters, size selection was performed. Illumina sequencing at a minimum coverage of 30× with 2 × 250 paired end sequencing was used to sequence the resulting fragments. The reads were trimmed using Trimmomatic^40^, to shorten the reads, and SAMTools^41^, and BED Tools were used to evaluate their quality. The raw sequence reads were assembled using SPAdes^42^. The assembled genomes were annotated on PATRIC (BV-BRC platform) and the genomic data was submitted to NCBI GenBank under the BioProject: PRJNA 985576, to get the accession numbers. The Average Nucleotide Identity (ANI) values for both the strains were calculated on EzBiocloud (https://www.ezbiocloud.net/) with a reference strain *Salmonella enterica* serovar Typhi (Taxonomy ID: 90370) retrieved from NCBI (https://www.ncbi.nlm.nih.gov/).

### Supplementary Information

Supplementary Information.

## Data Availability

The data sets generated and analyzed in this study (the whole genome sequence data of the strains MDR1-PH8 and XDR1-PG2) are available in NCBI (https://www.ncbi.nlm.nih.gov/), SRA (SUB13559018), under the BioProject: PRJNA985576, GenBank, (SUB13573411, SUB13573676) accession numbers JAUDSV000000000 and JAUDPT000000000, respectively. Similarly the 16S rDNA sequence data for the strains (PG2 and CM4) is available in NCBI Genbank with the accession numbers OP566900 (PG2) and OP604276 (CM4).
