# Supplementary material for: Prevalence and antibiotics resistance status of Salmonella in raw meat consumed in various areas of Lahore, Pakistan
Source: Sci Rep. 2023 Dec 14;13:22205. doi: 10.1038/s41598-023-49487-2 (PMC10721833; doi:10.1038/s41598-023-49487-2)
Supplement: Supplementary file 1 — Supplementary Information. [file 41598_2023_49487_MOESM1_ESM.pdf]

Prevalence and Antibiotics Resistance Status of *Salmonella* in Raw Meat Consumed in Various Areas of Lahore, Pakistan

Aiman Fatima‡, Maira Saleem‡, Shahid Nawaz‡, Linta Khalid‡, Saba Riaz‡, Imran Sajid‡,*

‡Institute of Microbiology and Molecular Genetics, University of the Punjab, Lahore-54590, Pakistan

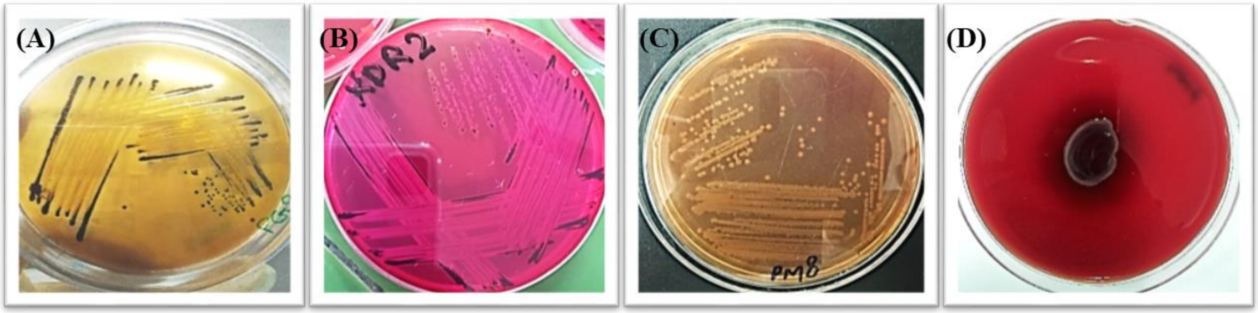

Figure S.1. Growth of *Salmonella* isolates on different media; **(A)** SS Agar **(B)** XLT-4 Agar **(C)** MacConkey Agar **(D)** Blood Agar.

Table S1. Oligonucleotide sequences of primers and PCR conditions used in this study.

Genes	Primers	Oligonucleotide Sequences (5'-3')	Product Size	Annealing Temp.	Extension Time	Reference
bla$_{\mathrm{TEM1}}$	Forward	AACCCTGGTAAATGCTTC	930bp	55ºC	1 min	Kim et al. 2021
	Reverse	GTATATATGAGTAAACTTGG				
catA1	Forward	GAAGATCACTTCGCAGAATAA	1003bp	45ºC	1 min	Kim et al. 2021
	Reverse	CAGCAATAGACATAAGCG				
gyrA	Forward	CTTTGAATCCGGGATACAG	2726bp	55ºC	2 min	Kim et al. 2021
	Reverse	TTCCATAGACAAGAAAAAGG				

Table S2. *Salmonella* load in retail raw meat sold at different local markets in Lahore, Pakistan.

Type of Sample	Total No. of Samples	No. of *Salmonella* (MPN/g or ml)			
		0.03-0.11	0.1-1.2	1.2-11	>11
Poultry meat	72	0	04	09	17
Goat meat	15	0	01	06	04
Buffalo meat	10	01	01	05	02
Cow meat	14	0	02	05	09
Total	111	01	08	25	32

Table S3. Antibiotics resistance pattern and resistance related genes in MDR and XDR *Salmonella* strains isolated from raw meat in Lahore, Pakistan.

**Antibiotic Resistance Pattern**	***Salmonella* Serovar**	**Phenotypic Resistance to Antibiotics**	**Antibiotic-Resistant Genes**	**Meat Source**
XDR	*S*. Typhi	AMP, C, SXT, CIP, LEV, CRO, CZ, AZM, IMP	$bla_{\text{TEM-1}}$, *catA1*, *gyrA*	Poultry gizzard
XDR	*S*. Typhimurium	AMP, C, SXT, CIP, LEV, CRO, CZ, AZM, IMP	$bla_{\text{TEM-1}}$, *catA1*, *gyrA*	Goat meat
XDR	*S*. Typhi	AMP, C, SXT, CIP, LEV, CRO, CZ, AZM, IMP	$bla_{\text{TEM-1}}$, *catA1*	Goat meat
MDR	*S*. Typhimurium	AMP, C, SXT, CIP, AZM, IMP	$bla_{\text{TEM-1}}$, *catA1*	Buffalo meat
MDR	*S*. Typhimurium	AMP, C, SXT, CIP, LEV, AZM, IMP	$bla_{\text{TEM-1}}$, *catA1*	Buffalo meat
MDR	*S*. Enteritidis	AMP, C, SXT, LEV, AZM, IMP	$bla_{\text{TEM-1}}$, *catA1*	Poultry heart
MDR	*S*. Typhimurium	AMP, C, SXT, CIP, AZM	$bla_{\text{TEM-1}}$, *catA1*	Poultry muscle
MDR	*S*. Typhi	AMP, C, SXT, CRO, CZ, AZM, IMP	$bla_{\text{TEM-1}}$	Poultry liver
MDR	*S*. Enteritidis	AMP, C, SXT, CIP, AZM, IMP	$bla_{\text{TEM-1}}$	Cow meat
MDR	*S*. Typhimurium	AMP, C, SXT, CRO, CZ, AZM	$bla_{\text{TEM-1}}$	Cow meat
MDR	*S. salamae*	AMP, C, SXT, CRO, AZM, IMP	$bla_{\text{TEM-1}}$	Goat meat
